# Case report and literature review: neuropsychiatric systemic lupus erythematosus presenting as massive intracerebral hemorrhage

**DOI:** 10.3389/fimmu.2026.1847320

**Published:** 2026-06-09

**Authors:** Yipeng Fang, Xin Kang, Anqi He, Jiayan Zhang, Junya Jia, Ying Gao, Keliang Xie

**Affiliations:** 1 Department of Critical Care Medicine, Tianjin Medical University General Hospital, Tianjin, China; 2 Department of Nephrology, Tianjin Medical University General Hospital, Tianjin, China

**Keywords:** antiphospholipid antibodies, case report, intracerebral hemorrhage, lupus anticoagulant, neuropsychiatric systemic lupus erythematosus

## Abstract

Neuropsychiatric systemic lupus erythematosus (NPSLE) encompasses a spectrum of central and peripheral nervous system manifestations. While cerebrovascular events occur in 5%-15% of NPSLE patients, intracerebral hemorrhage (ICH) as the initial presentation of systemic lupus erythematosus (SLE) is exceptionally rare and diagnostically challenging. A 17-year-old female presented with nephrotic syndrome and suddenly developed massive left frontal ICH requiring emergency craniotomy. Vascular malformations were excluded by digital subtraction angiography and intraoperative inspection. Subsequent immunological workup revealed a single positive antinuclear antibody, elevated anti-dsDNA antibodies, positive anti-ribosomal P antibody, positive lupus anticoagulant (LA), and hypocomplementemia, supporting the diagnosis of SLE with active lupus nephritis. She achieved multisystem recovery following aggressive immunosuppressive therapy. In the literature review, 12 clinical studies and 12 case reports were identified. The incidence of ICH was low (0.4%–1.5%), but the associated mortality risk was high (exceeding 20%). Compared with the non-SLE population, patients with SLE face a 2- to 3-fold higher risk of ICH. In case reports, all patients were female, 83.3% were aged ≤ 35 years, and 58.3% presented with ICH at the time of SLE diagnosis. Active lupus nephritis coexisted in 41.7% of cases. Common risk factors associated with the disease include young age, high disease activity, concomitant antiphospholipid syndrome, thrombocytopenia, and the use of antiplatelet agents. NPSLE should be considered in young female patients presenting with spontaneous ICH accompanied by multisystem involvement, particularly when conventional vascular risk factors are absent. The triad of active lupus nephritis, hypocomplementemia, and LA positivity may be associated with a high-risk phenotype for SLE-associated ICH. Timely recognition and appropriate immunomodulation may be important for improving outcomes.

## 1 Introduction

Systemic lupus erythematosus (SLE) is a chronic, multisystem autoimmune disease characterized by marked clinical heterogeneity and unpredictable disease flares (1). Neuropsychiatric SLE (NPSLE) represents one of the most severe disease manifestations, encompassing a broad spectrum ranging from subclinical neurocognitive dysfunction to life-threatening cerebrovascular events (2). Although cerebrovascular involvement occurs in only 5–15% of NPSLE cases, it confers disproportionately high morbidity and mortality (2, 3). Among SLE-related cerebrovascular disorders, ischemic stroke is the most common subtype; intracerebral hemorrhage (ICH) accounts for fewer than 20% of such events and has an overall incidence of less than 1% in large longitudinal SLE cohorts (3, 4). Given its rarity, abrupt clinical onset, and high case fatality rate, ICH constitutes one of the most catastrophic presentations of NPSLE. Female sex and age under 60 years have been robustly identified as independent risk factors for ICH in SLE patients (5). The pathogenesis of cerebrovascular events in NPSLE remains incompletely elucidated but is increasingly attributed to immune complex deposition, complement-mediated endothelial injury, and a prothrombotic state, which is often driven by antiphospholipid antibodies (aPL) (6).

ICH presenting as the initial manifestation of SLE, in patients with no prior diagnosis or conventional cardiovascular risk factors, is exceptionally rare and poses substantial diagnostic uncertainty. This scenario remains poorly documented in the literature, yet its recognition is clinically pivotal that timely identification shifts therapeutic priorities from isolated neurosurgical stabilization to urgent immunosuppressive therapy. We herein report a rare case of a young woman who initially presented with nephrotic syndrome and subsequently developed massive spontaneous ICH, after which further evaluation supported the diagnosis of SLE. In addition, we provide a literature review of published cases of SLE-associated ICH, synthesizing current evidence on its clinical phenotypes, key demographic and serological risk factors. This review aims to support earlier suspicion, accurate diagnosis, and prompt intervention in this rare but devastating complication.

## 2 Case description

### 2.1 History report

**Figure 1:**
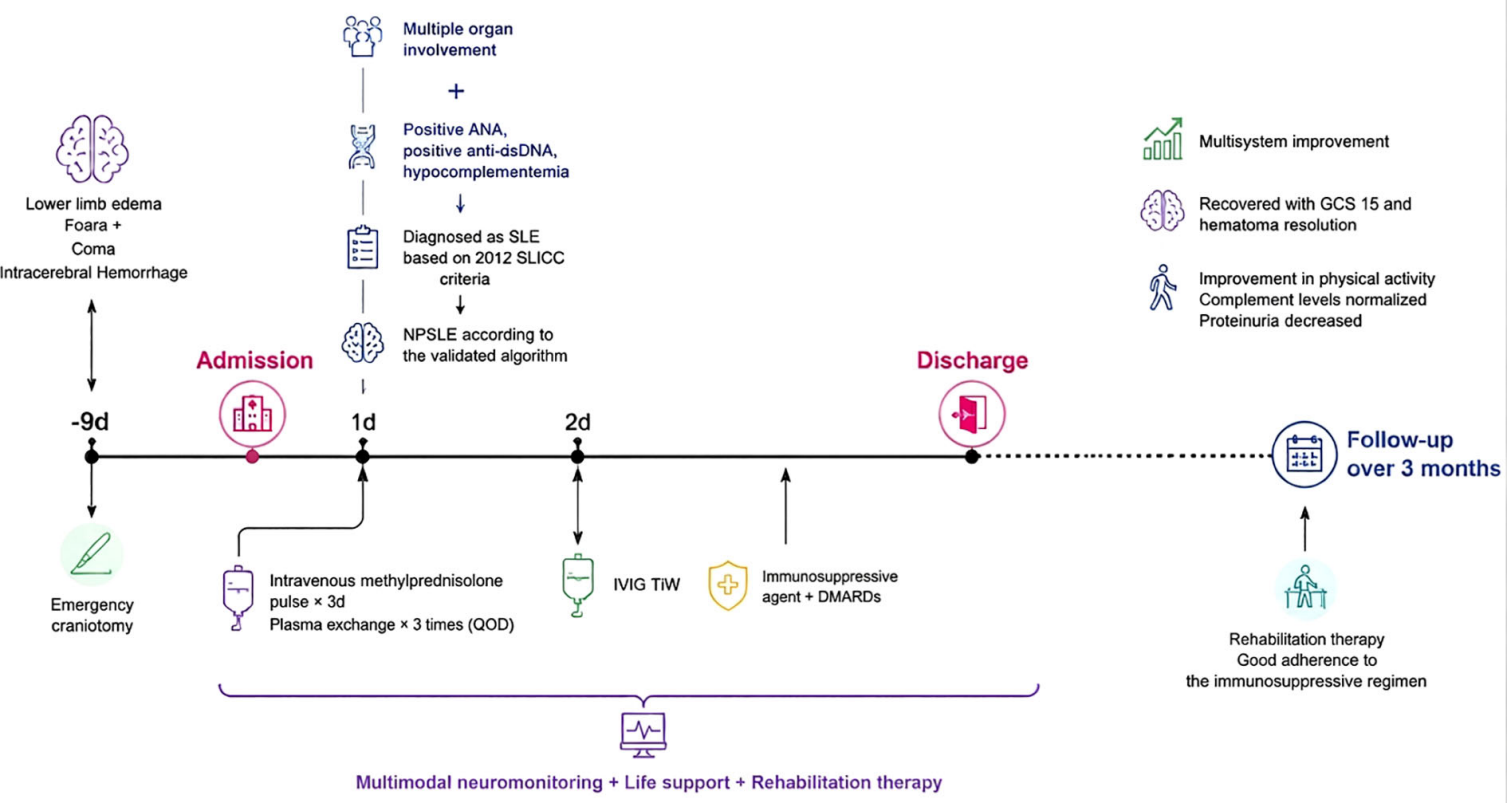
The timeline.

**Figure 2:**
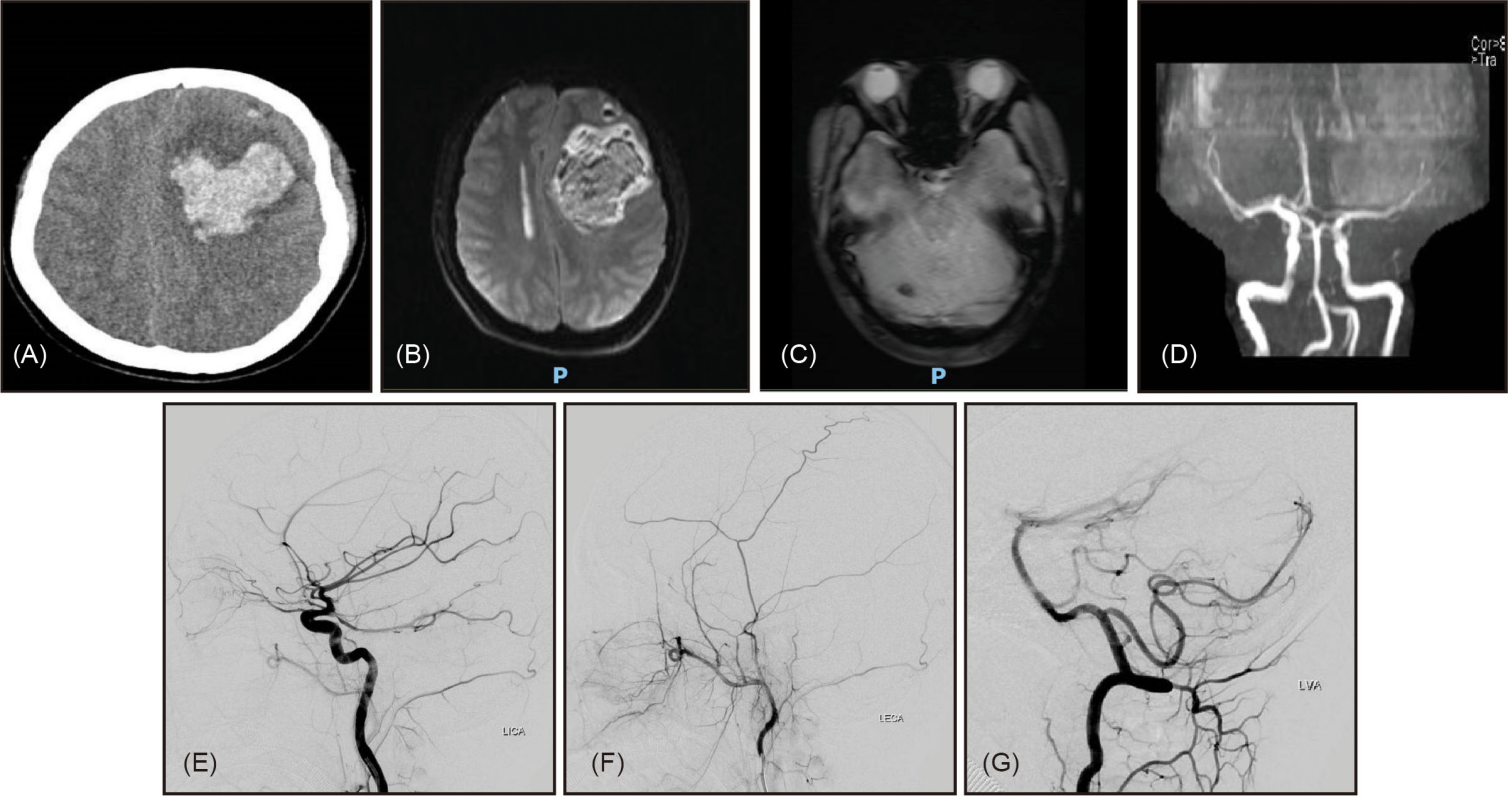
Emergency neuroimaging results of the patient. **(A, B)** Preoperative non-contrast **(A)** head CT and **(B)** brain MRI revealed intracerebral hemorrhage in the left cerebral hemisphere. **(C)** MRI also showed patchy GRE hypointensity with hemosiderin deposition in the right cerebellar hemisphere. **(D)** Preoperative magnetic resonance angiography revealed no evidence of aneurysm or arteriovenous malformation. **(E-G)** Preoperative emergency digital subtraction angiography of the left internal carotid artery **(E)**, left external carotid artery **(F)**, and left vertebral artery **(G)** similarly showed no evidence of aneurysm or arteriovenous malformation.

**Figure 3:**
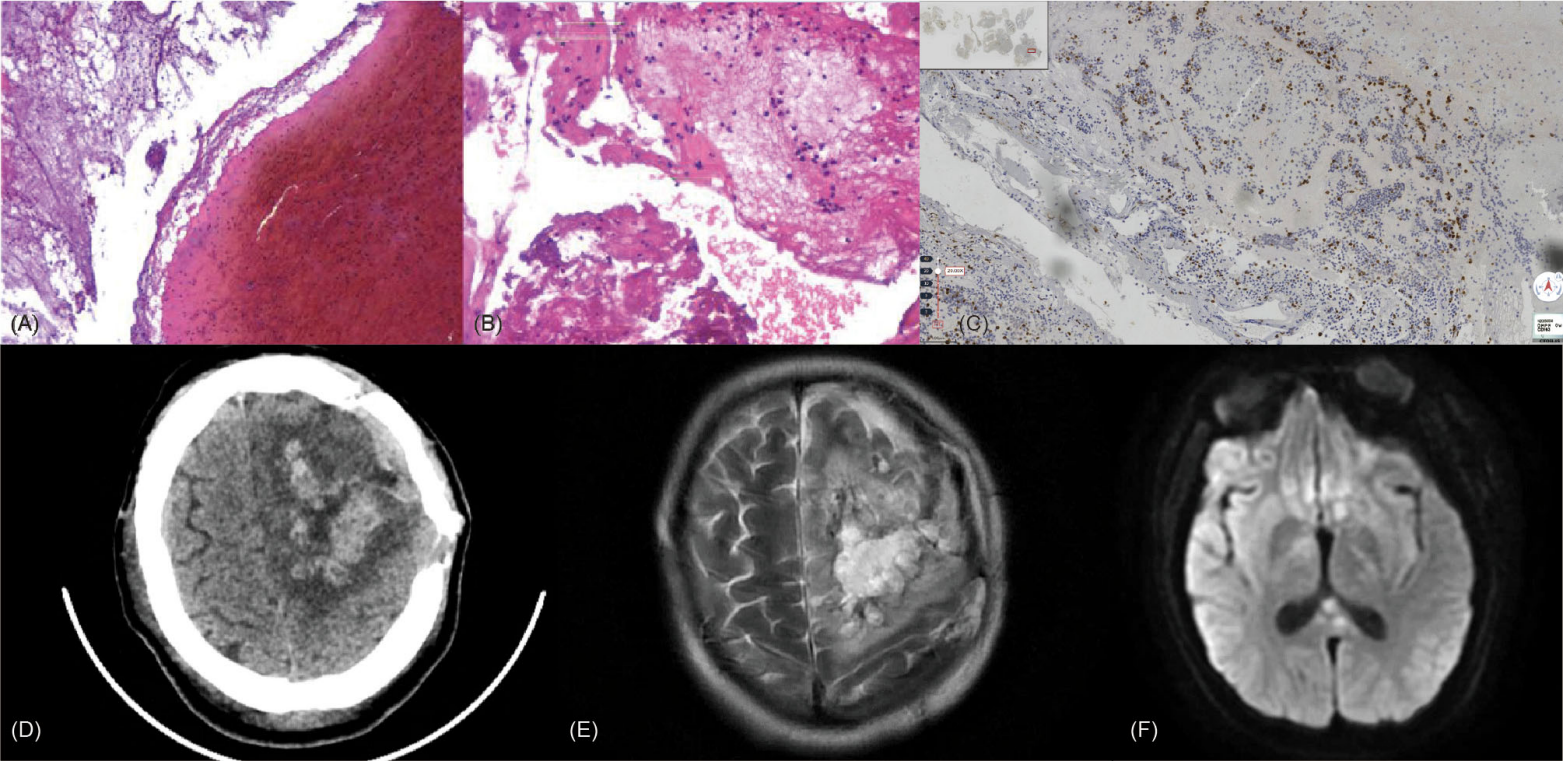
Postoperative histopathology and follow-up neuroimaging. **(A, B)** Intraoperative pathological findings. **(C)** Immunohistochemical staining for CD163. **(D)** Postoperative non-contrast head CT. **(E)** Postoperative brain MRI. **(F)** Diffusion-weighted imaging (DWI) demonstrating postoperative ischemic changes.

A 17-year-old female presented with a 9-day history of lower limb edema and foamy urine, raising suspicion of nephrotic syndrome (Timeline is presented in **Figure 1**). She denied recurrent headache, seizures, visual disturbances, or fever. During preoperative evaluation, she abruptly developed severe headache, vomiting, and slurred speech, rapidly progressing to coma. Emergency evaluation confirmed normal blood pressure and glucose. Cranial computerized tomography (CT) and magnetic resonance imaging (MRI) revealed a massive left frontal hematoma with intraventricular extension and midline shift (**Figure 2A, B**). MRI also demonstrated chronic microhemorrhagic foci in the right cerebellar hemisphere evidenced by hemosiderin deposits (**Figure 2C**), suggesting a background of diffuse microvasculopathy. Magnetic resonance angiography (MRA) and emergency digital subtraction angiography (DSA) excluded arteriovenous malformations or intracranial aneurysms (**Figure 2D-G**). Meanwhile, all dural venous sinuses were fully visualized, with no evidence of venous sinus thrombosis. Emergency craniotomy evacuated the hematoma, and intraoperative inspection confirmed the absence of vascular malformations. Histopathological examination of the perilesional tissue showed preserved microvascular architecture (**Figures 3A, B**) without overt vasculitis changes or necrosis. Scattered CD163-positive macrophages were present (**Figure 3C**), consistent with a reactive response to hemorrhage rather than primary vasculitis. Postoperatively, the patient remained comatose and received mechanical ventilation, osmotic therapy for intracranial pressure control, glucocorticoids, blood transfusions, and albumin support. For further treatment, the patient was transferred to our institution. The patient had been previously healthy, with no significant medical history, no long-term medication use, and no prior surgical interventions. Family history was unremarkable for autoimmune diseases or other hereditary conditions. On admission, neurological examination revealed a Glasgow Coma Scale score of 5+T (E4VTM1), left-sided anisocoria (left pupil 2.5 mm vs. right 2.0 mm), and a right-sided Babinski sign, which were consistent with acute left hemispheric injury. Vital signs were stable (blood pressure 126/83 mmHg, heart rate 91 bpm, respiratory rate 18/min, temperature 37.3 °C). Bilateral pulmonary crackles and pitting edema of the lower extremities were present.

### 2.2 Laboratory and imaging examination

Laboratory investigations revealed leukocytosis (17.64×10^9^/L with 80% neutrophils), normocytic anemia (hemoglobin 92 g/L), and a normal platelet count. Coagulation studies showed hyperfibrinogenemia (4.86 g/L) and markedly elevated D-dimer (3042 ng/mL). Biochemical profiling demonstrated hypoalbuminemia (21 g/L), hypertriglyceridemia (1.94 mmol/L), and low serum creatinine (36 μmol/L). Liver enzymes, electrolytes, and cardiac biomarkers were otherwise normal. Urinalysis confirmed nephrotic-range proteinuria (4+), microscopic hematuria (3+, 735 RBCs/μL with 90% dysmorphic), and granular casts (7.1/μL). The urine albumin-to-creatinine ratio was markedly elevated at 17,086 mg/g, with 24-hour urinary protein excretion ranging from 11 to 23 g. C-reactive protein was mildly elevated (1.14 mg/dL), while procalcitonin, β-D-glucan, and galactomannan were negative. Marked hypocomplementemia (C3 54.4 mg/dL, C4 5.98 mg/dL) was evident. Cerebrospinal fluid (CSF) analysis revealed a normal cell count, with a mildly elevated protein (0.45 g/L). CSF lactate dehydrogenase was elevated at 137.9 U/L. Both blood and CSF cultures were negative. Immunological evaluation revealed positive antinuclear antibody (ANA 1:320), elevated anti-dsDNA antibodies (236.4 U/mL), positive anti-C1q antibody (5.99 U/mL), positive anti-ribosomal P antibody, and a single positive lupus anticoagulant (LA, normalized dRVVT ratio 1.31, without confirmatory testing during hospitalization) while anticardiolipin and anti-β2-glycoprotein I antibodies were negative. Repeat neuroimaging demonstrated expected postoperative changes (**Figure 3D, E**) and new ischemic lesions in the deep white matter of the bilateral basal ganglia and frontal lobes, without imaging evidence of new cerebral hemorrhage (**Figure 3F**). Chest CT revealed small bilateral pleural effusions and a small pericardial effusion; abdominal CT identified free pelvic fluid without evidence of solid organ injury or intra-abdominal hemorrhage. Lower extremity venous ultrasound showed no evidence of deep vein thrombosis.

### 2.3 Diagnosis

Based on the 2012 SLICC criteria, the patient met five items: pleuritis, renal involvement, positive ANA, positive anti-dsDNA, and hypocomplementemia, establishing the diagnosis of SLE (7). The patient presented with coma and residual unilateral limb motor dysfunction following intracerebral hemorrhage but did not exhibit the more typical neuropsychiatric manifestations included in the 2012 SLICC criteria, such as seizures, psychosis, or acute confusional state. Lupus nephritis was clinically suspected given nephrotic syndrome, hematuria, and proteinuria up to 23 g/day (8); renal biopsy was deferred due to critical neuropsychiatric status.

To objectively assess attribution of ICH to NPSLE, we exploratorily attempted to apply the validated algorithm developed by Bortoluzzi et al., which generates a probability score ranging from 0 to 10 for neuropsychiatric events in SLE patients; scores greater than 6 suggest that the event is likely attributed to SLE (9). Our patient scored 10 points: (i) ICH occurring within 6 months of SLE diagnosis (3 points); (ii) absence of minor neuropsychiatric manifestations such as headache, anxiety, or mild cognitive complaints (3 points); (iii) no confounding comorbidities, including hypertension, diabetes mellitus, or anticoagulant/antiplatelet use (2 points); and (iv) presence of two SLE-specific risk factors: systemic disease activity manifesting as active lupus nephritis (LN) and laboratory-confirmed LA positivity (2 points). Although this maximal score does not establish causality, it provides strong support for a possible attribution of the ICH to NPSLE. The SLEDAI-2K score was 30, indicating severe, life-threatening disease activity.

### 2.4 Treatment and prognosis

An urgent immunosuppressive regimen was instituted: intravenous methylprednisolone pulse (500 mg daily for 3 days), followed by oral prednisone taper (starting at 40 mg/day) in combination with mycophenolate mofetil (500 mg twice daily) and hydroxychloroquine (200 mg daily). This was augmented by plasma exchange (2000 mL every other day for 3 sessions) and intravenous immunoglobulin (5 g twice weekly). Multimodal neuromonitoring and standard neurocritical care measures were implemented throughout the hospitalization. Although the patient was considered to have a high thrombotic risk, anticoagulation was deferred in the early phase due to the recent intracerebral hemorrhage and surgical management.

By the time of discharge, marked multisystem improvement was achieved. Neurologically, GCS had improved from 5 to 15 with speech recovery; follow-up imaging showed hematoma resolution. Renally, proteinuria decreased from 23 g/24h to 8.7 g/24h, while serum creatinine remained stable. Immunologically, complement levels normalized (C3 from 54.4 to 89 mg/dL; C4 from 5.98 to 16 mg/dL).

At the three-month follow-up, the patient remained clinically stable on maintenance immunosuppressive therapy. Laboratory assessment showed hemoglobin of 121 g/L, albumin 29.8 g/L; urinalysis revealed protein 1+, blood 1+, RBCs 20/μL, and no casts. The patient demonstrated good adherence to the immunosuppressive regimen, with no observed adverse effects or drug intolerance during the treatment course and follow-up period. She continues specialized neurorehabilitation for optimal functional recovery.

## 3 Literature review

**Figure 4:**
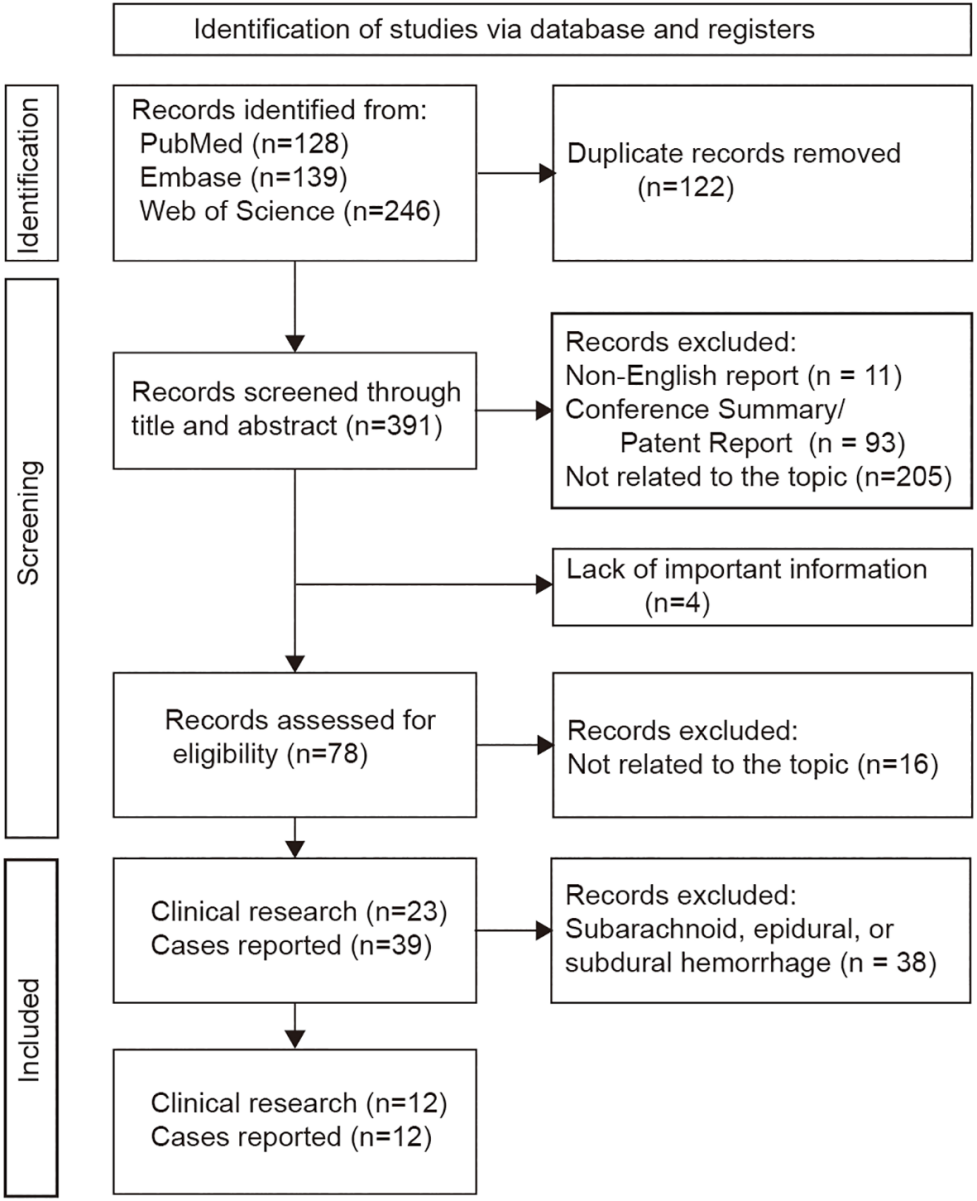
Flowchart of literature review.

Cerebrovascular events represent the fourth most common neuropsychiatric manifestation in NPSLE patients, with stroke being the predominant presentation (10). Although ischemic stroke predominates, ICH, while rare, carries disproportionately high mortality. Large cohort studies report ICH incidence in SLE patients ranging from 0.4% to 1.5%, with in-hospital mortality exceeding 20% in some series (4, 11, 12). Given its rarity and severity, SLE-related ICH remains poorly characterized, we therefore conducted a literature review of reported studies on SLE complicated by cerebral hemorrhage, published from 2005 to 2025 in the PubMed, Embase, and Web of Science databases, to better elucidate its clinical features, risk factors, and outcomes. The search strategy is available in the **Supplementary Table 1**. After removing duplicates, screening titles and abstracts, and evaluating full-text articles, we identified 62 eligible publications, comprising 23 clinical studies and 39 case reports (**Figure 4**). Details of the case reports are presented in **Supplementary Table 2**. After further excluding patients with subarachnoid, epidural, or subdural hemorrhage to focus on parenchymal bleeding, we ultimately identified 24 articles, resulting in 12 clinical studies and 12 case reports.

This review incorporates 12 clinical studies (shown in **Supplementary Table 3**), including one large case-control study, nine cohort studies, and two meta-analyses. Although ICH is relatively uncommon in SLE patients, multiple studies and two recent large meta-analyses confirm a 2–3 times higher ICH risk compared to non-SLE populations, with significantly elevated mortality (13–16). A Swedish nationwide cohort study documented 22% mortality in SLE patients with stroke versus 16% in non-SLE counterparts, with hemorrhagic strokes conferring a 2.3-fold increased death risk (17). Chinese data indicate an even more striking 23.1% in-hospital mortality for SLE patients with ICH, despite its low incidence of only 0.39% (4). Key risk factors for ICH in SLE patients include age, disease activity, disease duration, antiplatelet use, and thrombocytopenia. A Hong Kong cohort study demonstrated significantly increased ICH risk in young SLE patients compared to age-matched non-SLE individuals (18). Several large-scale Taiwan analyses identified critical predictors (19, 20). One study revealed a 3.2-fold higher stroke risk in SLE patients, with risk inversely correlating with age; the hazard ratio was highest in the youngest age group (≤ 17 years), hypertension and nephropathy emerged as the most significant comorbidity predictors (19). Another study highlighted disease activity’s pivotal role: when defining severe SLE flares by methylprednisolone pulse therapy requirement, hemorrhagic stroke incidence escalated from 0.4% in non-SLE controls to 1.3% in mild SLE and 2.7% in severe SLE (20). Furthermore, elevated SLEDAI-2K scores, hyperlipidemia, anemia, and APS were independently associated with increased hemorrhagic stroke risk in young SLE patients (16). The temporal relationship between SLE duration and ICH risk remains inconsistent across studies. A Taiwanese case-control study found 37% of hemorrhagic strokes occurred within the first year post-SLE diagnosis (16), whereas the Mexican cohort reported that >50% of hemorrhagic events occurred more than ten years after SLE diagnosis (11). A Swedish study reported a 2.5-fold higher ICH incidence during years 5–10 of SLE compared to the first 5 years (5). Notably, both latter studies excluded patients with pre-existing stroke at baseline. In addition, thrombocytopenia and antiplatelet use have been identified as associated factors for ICH in SLE patients. A large Taiwanese cohort study demonstrated that antiplatelet therapy is associated with an approximately 1.7-fold increased risk of hemorrhagic stroke (12). Furthermore, a Chinese cohort study revealed that thrombocytopenia is an independent risk factor for ICH in SLE patients, with an odds ratio of 3.687 (4).

In our review of 12 published cases of SLE-associated ICH (21–32) (see in **Table 1**), all patients were female (100%), with an age range from 18 to 60; notably, 83.3% (10/12) were aged 35 years or younger. The majority of ICH events occurred around the time of SLE diagnosis: 58.3% (7/12) presented with ICH as an initial manifestation of SLE. Structural lesions such as intracranial aneurysms were reported in only one case (29), and we excluded patients with subarachnoid, epidural, or subdural hemorrhage to focus on parenchymal bleeding. The most commonly identified etiologies were antiphospholipid syndrome (APS, 3 cases, 25.0%) (26, 30, 32), followed by central nervous system infection (22, 29), posterior reversible encephalopathy syndrome (21, 27), and severe thrombocytopenia (2 cases each, 16.7%) (23, 25). Other rare mechanisms included moyamoya syndrome and acquired factor XIII deficiency (one case each) (24, 31). Notably, active lupus nephritis was present in 41.7% (5/12) of patients (21, 22, 24, 27, 31), underscoring that ICH frequently arises in the context of high activity of lupus nephritis.

**Table 1:** Summary of case reports on SLE with ICH (2005–2025).

Author, year	Country	Patient characteristic	Aneurysm	cause of ICH	SLE course	Active lupus nephritis
Chen H, 2010 (21)	China, Taiwan	32y/Female	Non-reported	Posterior reversible encephalopathy syndrome	2 years	Yes
Bashir H, 2010 (22)	USA	52y/Female	Without	Central nervous system infection	30 years	Yes
Chattopadhyay P, 2011 (23)	India	24y/Female	Non-reported	Severe thrombocytopenia	Newly diagnosed	No
Wang R, 2013 (24)	China	24y/Female	Non-reported	Moyamoya syndrome	17 months	Yes
Pahadiya HR, 2016 (25)	India	35y/Female	Without	Severe thrombocytopenia	Newly diagnosed	No
Stavropoulos I, 2017 (26)	Greece	57y/Female	Without	APS	Newly diagnosed	No
Gauiran DTV, 2018 (27)	Philippines	33y/Female	Without	Posterior reversible encephalopathy syndrome	1 month	Yes
Yin R, 2019 (28)	China	43y/Female	Non-reported	Unknown	6 years	Unknown
Ueno M, 2019 (29)	Japan	60y/Female	With	Central nervous system infection	Newly diagnosed	No
Enescu CD, 2021 (30)	USA	18y/Female	Without	APS	Newly diagnosed	No
Aoki A, 2023 (31)	Japan	24y/Female	Without	Acquired factor XIII deficiency	Newly diagnosed	Yes
Khadka N, 2023 (32)	Nepal	18y/Female	Non-reported	APS	Newly diagnosed	No

APS, Antiphospholipid syndrome; ICH, Intracerebral Hemorrhage.

## 4 Discussion

We report a rare case of a 17-year-old female who presented initially with nephrotic syndrome and massive spontaneous ICH in the absence of hypertension, cerebral vascular malformations, or other conventional cerebrovascular risk factors. Neuroimaging demonstrated a characteristic triad, acute parenchymal hemorrhage, chronic cerebral microhemorrhages, and evolving ischemic lesions in deep gray and white matter, which, together with the systemic and immunological findings, supported a diagnosis of NPSLE with active lupus nephritis. Following prompt initiation of aggressive immunosuppressive therapy, the patient achieved substantial multisystem recovery, including neurological stabilization and renal remission. This case illustrates that delayed recognition of underlying SLE in young patients presenting with unexplained ICH may critically compromise therapeutic opportunity shifting management from isolated neurosurgical support to urgent, systemic immunomodulation as the cornerstone of care.

The etiology of ICH in this patient was challenging to determine. In young patients with ICH, structural vasculopathy is the most common cause; however, in our patient, vascular malformations, aneurysms, and other structural lesions were excluded by DSA and neuroimaging (33). Other recognized causes of ICH were also systematically evaluated. There was no histopathological evidence of cerebral vasculitis, no imaging evidence of cerebral venous sinus thrombosis, and no postoperative or recurrent bleeding to suggest a clinically significant coagulopathy, such as acquired factor XIII deficiency. Moreover, the patient did not exhibit features suggestive of PRES or RCVS, including headache, seizures, visual disturbances, vasogenic edema on MRI, or thunderclap headache (27, 34). Infectious aneurysm was also considered unlikely given the absence of fever, negative blood and cerebrospinal fluid cultures, absence of organisms on pathological examination, and normal angiographic findings (35). Taken together, structural, inflammatory, thrombotic, coagulopathic, vasoconstrictive, and infectious etiologies were considered less likely after comprehensive evaluation, thereby lending support to a diagnosis of SLE-associated ICH.

Attribution of ICH to NPSLE remains diagnostically challenging, because although neuropsychiatric manifestations occur in 17%-75% of SLE patients, they lack disease-specific biomarkers and exhibit substantial clinical and radiological overlap with primary neurological disorders (36). Using the validated attribution algorithm, which has demonstrated robust sensitivity and specificity, our patient achieved the maximal score of 10, which supports the possibility that NPSLE contributed importantly to the etiology of ICH (9, 37). However, it is important to note that this algorithm was developed and validated specifically in patients with an established SLE diagnosis who later developed neuropsychiatric events. In contrast, our patient received a diagnosis of SLE only after the ICH occurred. Therefore, applying this *post-hoc* attribution tool falls outside the original validation population and may introduce methodological bias. Accordingly, the scoring result for our patient is presented for clinical reference only.

NPSLE represents a heterogeneous syndrome resulting from SLE involvement of the nervous system, broadly categorized into diffuse and focal manifestations (2). Its pathogenesis is multifactorial, likely building upon a foundation of genetic susceptibility (6). Diffuse neuropsychiatric symptoms such as cognitive dysfunction, psychosis, and mood disorders are primarily attributed to blood-brain barrier (BBB) disruption, which permits circulating autoantibodies, particularly anti-ribosomal P antibodies, and inflammatory cytokines to enter the central nervous system, directly triggering excitotoxic neuronal injury, apoptosis, and synaptic dysfunction. Focal manifestations, most commonly cerebrovascular events, involve endothelial injury and complement activation. aPL activate endothelial cells, platelets, and monocytes, promoting cerebral thrombosis and accelerating atherosclerosis, leading to focal cerebral ischemia and intracranial vascular occlusion (38). Complement activation is closely linked to vascular wall inflammation, both amplifying aPL-mediated prothrombotic effects and independently compromising endothelial integrity (6). Although lupus anticoagulant and other antiphospholipid antibodies are classically associated with thrombosis, thrombotic and hemorrhagic mechanisms in NPSLE are not mutually exclusive. Immune-complex deposition, complement activation, and cytokine-mediated endothelial injury can induce diffuse cerebral microangiopathy, increase vascular permeability, and compromise small-vessel integrity (6). In parallel, aPL-mediated endothelial activation, platelet adhesion, and microthrombus formation may result in focal ischemia or microinfarction (39). Subsequent partial reperfusion of these ischemic regions may permit blood components to leak through the injured vascular wall into the brain parenchyma, leading to hemorrhagic transformation, a process further facilitated by blood-brain barrier breakdown and reperfusion injury (40). Therefore, intracerebral hemorrhage in NPSLE may arise not only from primary vessel fragility but also from secondary hemorrhagic conversion within an immune-mediated microangiopathic and prothrombotic milieu. Consequently, the cerebrovascular manifestations of NPSLE constitute a dynamic thrombo-ischemic-hemorrhagic continuum. In lupus nephritis, the relationship between renal injury and the abnormal activation of the complement system has been widely recognized (41). Our patient exhibited active lupus nephritis and unconfirmed LA positivity, suggesting that synergistic cerebrovascular injury may have been driven by LA-mediated thrombosis superimposed on complement-dependent endothelial damage during systemic disease flare. The neuroimaging triad, including acute hemorrhage, chronic microbleeds, and progressive ischemic infarcts, provides *in vivo* corroboration of this integrated pathobiology. Consistent with this mechanism, our review identified APS or aPL positive as the most frequent identifiable etiology of SLE-related ICH cases (25.0%) (26, 30, 32). Notably, NPSLE and active LN frequently co-occur, a postmortem study reported histopathological LN in 64.3% of NPSLE cases (42), and our review found concurrent LN in 41.7% of published ICH cases (21, 22, 24, 27, 31), reinforcing the possibility that SLE-related ICH typically arises in the context of high systemic inflammatory and thrombotic burden.

In this patient with suspected LA-positive SLE, the initial presentation was intracerebral hemorrhage, posing a clinically significant therapeutic dilemma: whether to initiate anticoagulation for suspected APS-related thrombosis or to delay it due to life-threatening active bleeding. Current guidelines recommend long-term vitamin K antagonist therapy for APS, with different INR targets depending on the presence or absence of venous thrombosis (43). For individuals with high-risk aPL profiles, including asymptomatic aPL carriers, patients with SLE without prior thrombotic or obstetric APS, and non-pregnant women with a history of obstetric APS only, low-dose aspirin is recommended (43). Additionally, for central nervous system involvement attributed to SLE, including cerebrovascular disease, early initiation of anticoagulation or antiplatelet therapy is advised (3). However, our patient was positive for LA on a single test only, with negative aPL and no clinical evidence of arterial or venous thrombosis. She did not meet the requirement of at least two positive aPL tests at least 12 weeks apart plus a thrombotic event (44). Therefore, a diagnosis of APS could not be formally established. Moreover, given that the patient presented with a large intracerebral hemorrhage as the initial symptom and underwent surgical intervention, the risk of hematoma expansion, rebleeding, and neurological deterioration was considered to outweigh the risk of thromboembolism. Guidelines for spontaneous intracerebral hemorrhage emphasize an individualized approach to antithrombotic therapy, balancing the risk of recurrent hemorrhage against thromboembolic risk. It is recommended to defer anticoagulation in the unstable acute phase after surgical intervention for large intracerebral hemorrhage, unless there is an urgent and compelling indication for anticoagulation (45). Accordingly, anticoagulation was not administered during the acute phase of hemorrhage in this case. In conclusion, a single positive LA test in patients with SLE-related intracerebral hemorrhage should prompt close monitoring for APS-related thrombosis; however, indiscriminate acute-phase anticoagulation should be avoided in the setting of hemodynamic instability following massive intracerebral hemorrhage or after surgical intervention.

Our patient tested positive for anti-ribosomal P antibodies, which target specific neuronal surface antigens and have been implicated in the pathogenesis of diffuse NPSLE manifestations (46). Although the present case predominantly manifested as focal cerebrovascular disease, the presence of this antibody underscores the potential for complex, overlapping pathogenic mechanisms that can coexist within the same patient.

CSF analysis revealed elevated protein with normal cell count and negative cultures. The origin of elevated CSF protein in NPSLE remains debated: it may reflect increased BBB permeability allowing peripheral proteins to enter the CNS or enhanced intrathecal immunoglobulin synthesis by resident immune cells (47). In our patient, the concurrent finding of chronic cerebral microhemorrhages on MRI, a marker of cumulative microvascular injury, suggests the possibility of underlying small-vessel vasculopathy (48). This vascular damage may be accompanied by BBB dysfunction, potentially facilitating entry of circulating autoantibodies (including anti-ribosomal P and LA) into the central nervous system, although direct evidence for BBB disruption in this case remains circumstantial. The mildly elevated CSF protein could reflect either increased BBB permeability or intrathecal antibody synthesis (49).

Several limitations warrant careful consideration. First, renal biopsy was deferred due to the patient’s critical neurological status that definitive histopathological classification of lupus nephritis could not be established. Nevertheless, the clinical and laboratory profile, including nephrotic-range proteinuria, active urinary sediment (hematuria with dysmorphic red blood cells and granular casts), and profound hypocomplementemia, was highly suggestive of proliferative lupus nephritis (ISN/RPS class III or IV). However, the magnitude of proteinuria (up to 23 g/day) also suggested the possibility of a concomitant membranous component (class V), consistent with possible mixed class III+V or IV+V lupus nephritis (8). Second, aPL testing was performed only once. Although LA positivity is a well-established contributor to cerebrovascular events in NPSLE, the single positive result in our patient does not fulfill the 2023 ACR/EULAR APS classification criteria, which require confirmatory testing after ≥12 weeks for formal APS classification (44). Therefore, while APS cannot be definitively diagnosed in this case, the presence of LA in the setting of a possible thrombotic event raises clinical suspicion for APS as an underlying mechanism contributing to SLE-associated ICH. Third, while the temporal, serological, and imaging evidence strongly implicates LA and complement activation as important potential contributors, the precise mechanistic hierarchy and relative contribution of each pathway cannot be definitively resolved in a single-case observation. Larger cohort studies and mechanistic investigations are needed to validate these findings. Fourth, the follow-up duration for this patient was limited, and the long-term prognosis and final outcome require further investigation.

## 5 Conclusion

The incidence of ICH as a manifestation of NPSLE is low, but the associated mortality risk is high. Common risk factors include young age, high disease activity, concomitant antiphospholipid syndrome, thrombocytopenia, and the use of antiplatelet agents. This case highlights that in the differential diagnosis of spontaneous ICH in young women, close attention should be paid to these risk factors, and NPSLE should be carefully considered, especially in the presence of multisystem involvement and the absence of conventional vascular risk factors. The triad of active lupus nephritis, hypocomplementemia, and LA positivity may represent a potentially high-risk clinical phenotype for SLE-associated ICH, likely reflecting convergent prothrombotic and complement-mediated endothelial injury. Timely diagnosis and early initiation of immunomodulatory therapy are not merely adjunctive measures but may be critical components of management and, in some cases, potentially life-saving. Given the rarity of this condition and the lack of prospective studies, this case report and literature review offer important reference for early clinical recognition and intervention.

## Data Availability

The raw data supporting the conclusions of this article will be made available by the authors, without undue reservation.

## References

[1] Thanou A Jupe E Purushothaman M Niewold TB Munroe ME . Clinical disease activity and flare in SLE: current concepts and novel biomarkers. J Autoimmun. (2021) 119:102615. doi: 10.1016/j.jaut.2021.102615 33631651 PMC8044029

[2] The American College of Rheumatology . Nomenclature and case definitions for neuropsychiatric lupus syndromes. Arthritis Rheum. (1999) 42:599–608. doi: 10.1002/1529-0131(199904)42:4<599::AID-ANR2>3.0.CO;2-F 10211873

[3] Bertsias GK Ioannidis JP Aringer M Bollen E Bombardieri S Bruce IN . EULAR recommendations for the management of systemic lupus erythematosus with neuropsychiatric manifestations: report of a task force of the EULAR standing committee for clinical affairs. Ann Rheum Dis. (2010) 69:2074–82. doi: 10.1136/ard.2010.130476 20724309

[4] Gao N Wang ZL Li MT Han SM Dang YQ Zhang FC . Clinical characteristics and risk factors of intracranial hemorrhage in systemic lupus erythematosus. Lupus. (2013) 22:453–60. doi: 10.1177/0961203313477226 23554034

[5] Arkema EV Svenungsson E Von Euler M Sjöwall C Simard JF . Stroke in systemic lupus erythematosus: a Swedish population-based cohort study. Ann Rheum Dis. (2017) 76:1544–9. doi: 10.1136/annrheumdis-2016-210973 28400384

[6] Liu Y Tu Z Zhang X Du K Xie Z Lin Z . Pathogenesis and treatment of neuropsychiatric systemic lupus erythematosus: a review. Front Cell Dev Biol. (2022) 10:998328. doi: 10.3389/fcell.2022.998328 36133921 PMC9484581

[7] Petri M Orbai AM Alarcón GS Gordon C Merrill JT Fortin PR . Derivation and validation of the Systemic Lupus International Collaborating Clinics classification criteria for systemic lupus erythematosus. Arthritis Rheum. (2012) 64:2677–86. doi: 10.1002/art.34473 22553077 PMC3409311

[8] Kidney Disease: Improving Global Outcomes (KDIGO) Lupus Nephritis Work Group . KDIGO 2024 clinical practice guideline for the management of LUPUS NEPHRITIS. Kidney Int. (2024) 105:S1–S69. doi: 10.1016/j.kint.2023.09.002 38182286

[9] Bortoluzzi A Scirè CA Bombardieri S Caniatti L Conti F De Vita S . Development and validation of a new algorithm for attribution of neuropsychiatric events in systemic lupus erythematosus. Rheumatol (Oxford). (2015) 54:891–8. doi: 10.1093/rheumatology/keu384 25339643

[10] Hanly JG Li Q Su L Urowitz MB Gordon C Bae SC . Cerebrovascular events in systemic lupus erythematosus: results from an international inception cohort study. Arthritis Care Res (Hoboken). (2018) 70:1478–87. doi: 10.1002/acr.23509 29316357 PMC6033693

[11] Guraieb-Chahín P Cantú-Brito C Soto-Mota A Guerrero-Torres L Flores-Silva F Chiquete E . Stroke in systemic lupus erythematosus: epidemiology, mechanism, and long-term outcome. Lupus. (2020) 29:437–45. doi: 10.1177/0961203320908947 32151182

[12] Huang JA Lin CH Wu MJ Chen YH Chang KC Hou CW . Ten-year follow-up investigation of stroke risk in systemic lupus erythematosus. Stroke Vasc Neurol. (2024) 9:1–7. doi: 10.1136/svn-2022-001499 37169398 PMC10956114

[13] Yazdany J Pooley N Langham J Nicholson L Langham S Embleton N . Systemic lupus erythematosus; stroke and myocardial infarction risk: a systematic review and meta-analysis. RMD Open. (2020) 6:e001247. doi: 10.1136/rmdopen-2020-001247 32900883 PMC7722272

[14] Holmqvist M Simard JF Asplund K Arkema EV . Stroke in systemic lupus erythematosus: a meta-analysis of population-based cohort studies. RMD Open. (2015) 1:e000168. doi: 10.1136/rmdopen-2015-000168 26719816 PMC4692049

[15] Bernatsky S Clarke A Gladman DD Urowitz M Fortin PR Barr SG . Mortality related to cerebrovascular disease in systemic lupus erythematosus. Lupus. (2006) 15:835–9. doi: 10.1177/0961203306073133 17211987

[16] Hsu UH Lin YT Chiang BL . The characteristics and risk factors of cerebrovascular events in young systemic lupus erythematosus patients: a case-control study. J Formos Med Assoc. (2024) 123:478–86. doi: 10.1016/j.jfma.2023.09.018 37813767

[17] Rossides M Simard JF Svenungsson E von Euler M Arkema EV . Mortality and functionality after stroke in patients with systemic lupus erythematosus. J Rheumatol. (2017) 44:1590–6. doi: 10.3899/jrheum.170241 28916550

[18] Mok CC Ho LY To CH . Annual incidence and standardized incidence ratio of cerebrovascular accidents in patients with systemic lupus erythematosus. Scand J Rheumatol. (2009) 38:362–8. doi: 10.1080/03009740902776927 19296403

[19] Wang IK Muo CH Chang YC Liang CC Lin SY Chang CT . Risks, subtypes, and hospitalization costs of stroke among patients with systemic lupus erythematosus: a retrospective cohort study in Taiwan. J Rheumatol. (2012) 39:1611–8. doi: 10.3899/jrheum.111510 22753653

[20] Chang KC Lin CH Chen PL Wu YH Hou CW Huang JA . Severe lupus flare is associated with a much higher risk of stroke among patients with SLE. Int J Stroke. (2023) 18:957–64. doi: 10.1177/17474930231174227 37089085

[21] Chen HA Lin YJ Chen PC Chen TY Lin KC Cheng HH . Systemic lupus erythematosus complicated with posterior reversible encephalopathy syndrome and intracranial vasculopathy. Int J Rheum Dis. (2010) 13:e79. doi: 10.1111/j.1756-185X.2010.01545.x 21199460

[22] Bashir H Ranganathan P . An unusual case of cerebellar hemorrhage in a patient with systemic lupus erythematosus. Arthritis Care Res (Hoboken). (2010) 62:738–42. doi: 10.1002/acr.20114 20191476

[23] Chattopadhyay P Dhua D Philips C . Reversible diffuse neurological deficits in systemic lupus erythematosus: report of a case. Lupus. (2011) 20:1079–85. doi: 10.1177/0961203310396268 21478287

[24] Wang R Xu Y Lv R Chen J . Systemic lupus erythematosus associated with Moyamoya syndrome: a case report and literature review. Lupus. (2013) 22:629–33. doi: 10.1177/0961203313485828 23574743

[25] Pahadiya HR Lakhotia M Gandhi R Choudhary A Madan S . Multiple intracranial hemorrhages in pregnancy: a common autoimmune etiology. J Neurosci Rural Pract. (2016) 7:290–4. doi: 10.4103/0976-3147.178663 27114665 PMC4821942

[26] Stavropoulos I Liverezas A Papageorgiou E Tsiara S . A rare case of heparin-induced thrombocytopenia and cerebral venous sinus thrombosis with antiphospholipid syndrome and possible systemic lupus erythematosus. Aktual Neurol. (2017) 17:121. doi: 10.15557/AN.2017.0013

[27] Gauiran D Lladoc-Natividad T Rocha I Manapat-Reyes BH . Seizure and acute vision loss in a Filipino lupus patient: a case of posterior reversible encephalopathy syndrome with intraparenchymal hemorrhage. Case Rep Med. (2018) 2018:4238676. doi: 10.1155/2018/4238676 30631368 PMC6304919

[28] Yin R Qiu C-X Yin L-J Zhang Y . Case report multiple spontaneous intracranial hemorrhages in a patient with systemic lupus erythematosus: a case report. J Clin Exp Med. (2019) 12:10900.

[29] Ueno M Nakano K Yoshinari H Nakayamada S Iwata S Kubo S . An autopsy case with cerebral hemorrhaging due to disseminated aspergillosis during glucocorticoid therapy for overlap syndrome of systemic lupus erythematosus and systemic sclerosis. Intern Med. (2019) 58:1023–7. doi: 10.2169/internalmedicine.1226-18 30568120 PMC6478969

[30] Enescu CD Basida B Zalavadiya N Akram R Sarakbi H . A diagnostic dilemma: catastrophic or seronegative antiphospholipid syndrome. Cureus. (2021) 13:e18745. doi: 10.7759/cureus.18745 34790491 PMC8588193

[31] Aoki A Kobayashi H . Systemic lupus erythematosus with cerebral hemorrhage due to autoimmune acquired coagulation FXIII/13 factor deficiency: a case study and literature review. Ann Rheum Dis. (2023) 82:1499. doi: 10.1136/annrheumdis-2023-eular.651 37280048

[32] Khadka N Kandel K Mishra A Jha S . Concurrent occurrence of acute pancreatitis and intracerebral hemorrhage as presenting manifestations in lupus: a case report. Ann Med Surg (Lond). (2023) 85:4067–70. doi: 10.1097/MS9.0000000000001009 37554859 PMC10406074

[33] Chen CY Lin PT Wang YH Syu RW Hsu SL Chang LH . Etiology and risk factors of intracranial hemorrhage and ischemic stroke in young adults. J Chin Med Assoc. (2021) 84:930–6. doi: 10.1097/JCMA.0000000000000598 34380990 PMC12966178

[34] Avola G Pezzini A . Treatment-related reversible cerebral vasoconstriction syndrome. J Clin Med. (2024) 13:5930. doi: 10.3390/jcm13195930 39407990 PMC11478140

[35] Rangwala SD Strickland BA Rennert RC Ravina K Bakhsheshian J Hurth K . Ruptured mycotic aneurysm of the distal circulation in a patient with mucormycosis without direct skull base extension: case report. Oper Neurosurg (Hagerstown). (2019) 16:E101–7. doi: 10.1093/ons/opy127 29800469

[36] Li J Meng H Jiang W Liu J Cui Z Miao J . Cerebral venous sinus thrombosis and subdural hematoma in a female patient with systemic lupus erythematosus: a case report and literature review. Ann Palliat Med. (2021) 10:8454–9. doi: 10.21037/apm-20-2285 33977732

[37] Magro-Checa C Zirkzee EJ Beaart-van de Voorde LJJ Middelkoop HA van der Wee NJ Huisman MV . Value of multidisciplinary reassessment in attribution of neuropsychiatric events to systemic lupus erythematosus: prospective data from the Leiden NPSLE cohort. Rheumatol (Oxford). (2017) 56:1676–83. doi: 10.1093/rheumatology/kex019 28339952

[38] Meier AL Bodmer NS Wirth C Bachmann LM Ribi C Pröbstel AK . Neuro-psychiatric manifestations in patients with systemic lupus erythematosus: a systematic review and results from the Swiss lupus cohort study. Lupus. (2021) 30:1565–76. doi: 10.1177/09612033211025636 34152246 PMC8489688

[39] Raschi E Borghi MO Tedesco F Meroni PL . Antiphospholipid syndrome pathogenesis in 2023: an update of new mechanisms or just a reconsideration of the old ones. Rheumatol (Oxford). (2024) 63:SI4–SI13. doi: 10.1093/rheumatology/kead603 38320591

[40] Zubair AS Sheth KN . Hemorrhagic conversion of acute ischemic stroke. Neurotherapeutics. (2023) 20:705–11. doi: 10.1007/s13311-023-01377-1 37085684 PMC10275827

[41] Anders HJ Saxena R Zhao MH Parodis I Salmon JE Mohan C . Lupus nephritis. Nat Rev Dis Primers. (2020) 6:7. doi: 10.1038/s41572-019-0141-9 31974366

[42] Carrión-Barberà I Salman-Monte TC Vílchez-Oya F Monfort J . Neuropsychiatric involvement in systemic lupus erythematosus: a review. Autoimmun Rev. (2021) 20:102780. doi: 10.1016/j.autrev.2021.102780 33609799

[43] Tektonidou MG Andreoli L Limper M Amoura Z Cervera R Costedoat-Chalumeau N . EULAR recommendations for the management of antiphospholipid syndrome in adults. Ann Rheum Dis. (2019) 78:1296–304. doi: 10.1136/annrheumdis-2019-215213 31092409 PMC11034817

[44] Barbhaiya M Zuily S Naden R Hendry A Manneville F Amigo MC . 2023 ACR/EULAR antiphospholipid syndrome classification criteria. Ann Rheum Dis. (2023) 82:1258–70. doi: 10.1136/ard-2023-224609 37640450

[45] Greenberg SM Ziai WC Cordonnier C Dowlatshahi D Francis B Goldstein JN . 2022 guideline for the management of patients with spontaneous intracerebral hemorrhage: a guideline from the American Heart Association/American Stroke Association. Stroke. (2022) 53:e282–361. doi: 10.1161/STR.0000000000000407 35579034

[46] Manca E . Autoantibodies in neuropsychiatric systemic lupus erythematosus (NPSLE): can they be used as biomarkers for the differential diagnosis of this disease. Clin Rev Allergy Immunol. (2022) 63:194–209. doi: 10.1007/s12016-021-08865-2 34115263 PMC9464150

[47] Schwartz N Stock AD Putterman C . Neuropsychiatric lupus: new mechanistic insights and future treatment directions. Nat Rev Rheumatol. (2019) 15:137–52. doi: 10.1038/s41584-018-0156-8 30659245 PMC8023338

[48] Jolink WMT van Veluw SJ Zwanenburg JJM Rozemuller AJM van Hecke W Frosch MP . Histopathology of cerebral microinfarcts and microbleeds in spontaneous intracerebral hemorrhage. Transl Stroke Res. (2023) 14:174–84. doi: 10.1007/s12975-022-01016-5 35384634 PMC9995541

[49] Moore E Huang MW Putterman C . Advances in the diagnosis, pathogenesis and treatment of neuropsychiatric systemic lupus erythematosus. Curr Opin Rheumatol. (2020) 32:152–8. doi: 10.1097/BOR.0000000000000682 31895125 PMC7548105

