# Supplementary material for: Case report and literature review: neuropsychiatric systemic lupus erythematosus presenting as massive intracerebral hemorrhage
Source: Front Immunol. 2026 Jun 9;17:1847320. doi: 10.3389/fimmu.2026.1847320 (PMC13286940; doi:10.3389/fimmu.2026.1847320)
Supplement: Supplementary file 1 [file Table1.docx]

**Table S1** The search terms for literature review using in PubMed, Embase, and Web of Science databases from 2005 to 2025

| Database | SAearch terms |
| --- | --- |
| Pubmed | ("Lupus Erythematosus, Systemic"[Mesh] OR "Lupus Nephritis"[Mesh] OR "Lupus Vasculitis, Central Nervous System"[Mesh] OR "systemic lupus erythematosus"[tiab] OR SLE[tiab] OR "Lupus Vasculitis"[tiab] OR "Lupus Nephritis"[tiab]) AND ("Cerebral Hemorrhage"[Mesh] OR "Intracranial Hemorrhages"[Mesh] OR "cerebral hemorrhage*"[tiab] OR "brain hemorrhage*"[tiab] OR "intracranial hemorrhage*"[tiab] OR "intracerebral hemorrhage*"[tiab] OR "Cerebral Parenchymal Hemorrhage*"[tiab] OR "ICH"[tiab]) |
| Embase | (('systemic lupus erythematosus'/de OR 'lupus cerebritis'/de) AND ('intracerebral hemorrhage'/de OR 'brain hemorrhage'/de OR 'cerebral hemorrhage'/de)) AND ((('systemic lupus erythematosus' OR 'SLE' OR 'lupus cerebritis'):ti) OR (('systemic lupus erythematosus' OR 'SLE' OR 'lupus cerebritis'):ab AND ('intracerebral hemorrhage' OR 'cerebral hemorrhage' OR 'brain hemorrhage' OR 'ICH'):ab)) AND [2005-2025]/py AND [humans]/lim AND ('article'/it OR 'review'/it) |
| Web of science | TS=("systemic lupus erythematosus" OR "lupus erythematosus, systemic" OR "SLE" OR "systemic lupus erythematosus disease") AND TS=("intracerebral hemorrhage" OR "intracerebral haemorrhage" OR "cerebral hemorrhage" OR "cerebral haemorrhage" OR "brain hemorrhage" OR "brain haemorrhage" OR "intraparenchymal hemorrhage" OR "intraparenchymal haemorrhage" OR "ICH") AND PY=(2005-2025) |
