# Supplementary material for: Case report and literature review: neuropsychiatric systemic lupus erythematosus presenting as massive intracerebral hemorrhage
Source: Front Immunol. 2026 Jun 9;17:1847320. doi: 10.3389/fimmu.2026.1847320 (PMC13286940; doi:10.3389/fimmu.2026.1847320)
Supplement: Supplementary file 2 [file Table2.docx]

**Table S2.** Summary of Case Reports on SLE with CNS Hemorrhage (2005-2025)

| **Author, Year** | **Country/Region** | **Patient Characteristic** | **Aneurysm** | **Type of hemorrhage** | **Comorbidities** | **SLE course** |
| --- | --- | --- | --- | --- | --- | --- |
| Misra, DP, 2015 (1) | India | 39y/Female | With | SAH | Protein S functional deficiency, dural sinus thrombosis | Unknown |
| Argyrou A, 2010 (2) | Greece | 17y/Female | Non-reported | Unknown | Noonan syndrome, TTP | Unknown |
| Chen H, 2010 (3) | China, Taiwan | 32y/Female | Non-reported | ICH + SAH | LN, PRES | 2 years |
| Yazdany J .2020 (4) | Taiwan | 39y/Female | Non-reported | SAH | N/A | Unknown |
| Wang R, 2013 (5) | China | 24y/Female | Non-reported | ICH | MMS | 10 months |
| Kozak HH, 2014 (6) | Turkey | 43 y/Female | Without | SAH | Renal hypertension | 10 years |
| Li N, 2024 (7) | China | 52y/Female | Without | ICH | N/A | 27 years |
| Khorolsky C,2018 (8) | USA | 40y/Female | With | SAH | Hypertension, SLE–AAV overlap syndrome | newly-diagnosed |
| Wen Y, 2021 (9) | China | 12y/Female | Non-reported | SDH | TTP | newly-diagnosed |
| Santacruz JC, 2023 (10) | Colombia | 57y/Male | Non-reported | ICH | Severe pancytopenia | newly-diagnosed |
| Torné R ,2015 (11) | Spain | 34y/Male + 24y/Female+ 55y/Male | 2 with | SAH | LN, renal hypertension | Non-reported |
| Owada T ,2009 (12) | Japan | 51y/Female+ 43y/Female | With | SAH | LN, TTP | newly-diagnosed |
| Zhang Y ,2019 (13) | China | 21y/Female | With | SAH | N/A | 1 year |
| Tsukamoto E, 2020 (14) | Japan | 22y/Male | With | SAH | APS | newly-diagnosed |
| Song W, 2015 (15) | China | 29y/Female | Without | EDH | N/A | 1 year |
| Gauiran DTV, 2018 (16) | Philippines | 33y/Female | Without | ICH | LN, PRES, hypertension, Thrombocytopenia | 1 month |
| Aoki A, 2023 (17) | Japan | 24y/Female | Without | ICH | Acquired coagulation factor XIII deficiency | newly-diagnosed |
| Rangwala SD, 2019 (18) | USA | 27y/Female | With | SAH | Aspergillus fungal aneurysm rupture | Unknown |
| Chattopadhyay P, 2011 (19) | India | 24y/Female | Non-reported | ICH+SAH | Thrombocytopenia | newly-diagnosed |
| Ramagopalan SV, 2013 (20) | Malaysia | 22y/Female | Non-reported | SDH | Immune thrombocytopenia and overlap syndrome. | 2 years |
| Yin R, 2019 (21) | China | 43y/Female | Non-reported | EDH + ICH | N/A | Unknown |
| Pahadiya HR, 2016 (22) | India | 35y/Female | Without | ICH | Thrombocytopenia | newly-diagnosed |
| Carreño-Tarragona G, 2016 (23) | Bulgaria | 37y/Male | Without | SDH | LAHS, Anticoagulation | 11 year |
| Llorente-Chávez, 2021 (24) | Mexico | 50y/Female | Non-reported | SDH | LAHS, acquired hemophilia | Unknown |
| Boura P, 2005 (25) | Greece | 31y/Female | Non-reported | Unknown | CAPS, Anticoagulant and antiplatelet therapy | newly-diagnosed |
| Perard L, 2007 (26) | France | 22y/Female | Without | Unknown | Thrombocytopenia, hypertension, eclampsia | 2 years |
| Lee JH,2020 (27) | Korea | 44y/Female | With | Contrast agent extravasation | Antiplatelet therapy | 17 years |
| Khadka N, 2023 (28) | Nepal | 18y/Female | Non-reported | ICH | APS, hypertension | newly-diagnosed |
| Li J, 2021 (29) | China | 44y/Female | Non-reported | SDH | CVST, Thrombocytopenia | newly-diagnosed |
| Casanova Rivera MF, 2023 (30) | Ecuador | 17y/Female | Without | ICH | CVST, APS | newly-diagnosed |
| Yin R, 2019 (31) | China | 45y/Female | Non-reported | EDH | N/A | Unknown |
| Harriott A, 2016 (32) | USA | 42y/Female | With | SAH | N/A | Unknown |
| Bashir H, 2010 (33) | USA | 52y/Female | Without | ICH | CNS Nocardiosis | 30 years |
| Ueno M, 2019 (34) | Japan | 60y/Female | With | ICH | Overlapping syndrome, aspergillosis, thrombocytopenia | newly-diagnosed |
| Sandouno TM, 2021 (35) | Morocco | 43y/Female | Without | SDH | Acquired coagulation factor XIII deficiency | newly-diagnosed |
| Stavropoulos I, 2017 (36) | Greece | 57y/Female | Without | ICH | Anticoagulation, hypertension, thrombocytopenia, CVST | newly-diagnosed |
| Hui SC ,2023 (37) | China | 79y/Female | Non-reported | ICH | Pancytopenia, lung cancer | newly-diagnosed |
| Enescu CD, 2021 (38) | USA | 18y/Female | Without | ICH | Seronegativ CAPS | newly-diagnosed |
| Okumura E, 2023 (39) | Japan | 24y/Female | Without | ICH | Acquired coagulation factor XIII deficiency | newly-diagnosed |
| Wu R, 2017 (40) | China | 15y/Female | Without | ICH | CVST, Thrombocytopenia | newly-diagnosed |

**Abbreviations: AAV, Antineutrophil cytoplasmic antibody-associated vasculitis; APS, Antiphospholipid syndrome; CAPS, Catastrophic antiphospholipid syndrome; CVST, Cerebral venous sinus thrombosis; EDH, Epidural Hematoma; ICH, Intracerebral Hemorrhage; LAHS, Lupus anticoagulant-hypoprothrombinemia syndrome; LN, Lupus nephritis; MMS,** **Moyamoya syndrome; PRES, Posterior reversible encephalopathy syndrome; SAH, Subarachnoid Hemorrhage; SDH, Subdural Hematoma; TTP, Thrombotic thrombocytopenic purpura.**

**References**

1. Misra DP, Chowdhury AC, Parida JR, Jain SK, Agarwal V. Unusual co-existence of dural sinus thrombosis and aneurysmal subarachnoid hemorrhage in a patient with lupus. *Lupus* (2015) 24(9):994-7. doi: 10.1177/0961203315570687

2. Argyrou A, Marinakis T, Kalofolias N, Papazoglou S, Anagnostopoulos NI. Thrombotic thrombocytopenic purpura in a young patient with Noonan syndrome and systemic lupus erythematosus. *Archives of Hellenic MedicineArch. Hell. Med.* (2010) 27(3):545.

3. Chen H, Lin Y, Chen P, Chen T, Lin K, Cheng H. Systemic lupus erythematosus complicated with posterior reversible encephalopathy syndrome and intracranial vasculopathy. *Int J Rheum Dis* (2010) 13(4):e79-79e82. doi: 10.1111/j.1756-185X.2010.01545.x

4. Tang S-C, Lee C-F, Lee C-W, Jeng J-S. Systemic lupus erythematosus flare up manifestation as cerebral and spinal subarachnoid hemorrhage. *LupusLupus* (2011) 20(11):1211. doi: 10.1177/0961203311399305

5. Wang R, Xu Y, Lv R, Chen J. Systemic lupus erythematosus associated with moyamoya syndrome: A case report and literature review. *LupusLupus* (2013) 22(6):629. doi: 10.1177/0961203313485828

6. Kozak HH, Çorbacıoğlu MB, Anlıaçık SÖ. Systemic lupus erythematosus and subarachnoid hemorrhage. *Neurol India* (2014) 62(6):689-90.

7. Li N, Liu YT, Tao CY. Systemic lupus erythematosus and antiphospholipid syndrome complicating infratentorial subdural hematoma: A case report. *Asian J Surg* (2024) 47(4):1869-70.

8. Khorolsky C, 0000-0002-1062-9131 AO, Castellano A, 0000-0002-1038-0647 AO, Comstock D, 0000-0003-3657-237X AO, et al. Systemic lupus erythematosus and antineutrophilic cytoplasmic antibody-associated vasculitis overlap syndrome complicated by subarachnoid hemorrhage: case-based review. *Rheumatol Int* (2018) 38(12):2329-35.

9. Wen Y, Lu P, Lu H, Hu X. Successful treatment of subdural hemorrhage and retinal hemorrhage in childhood-onset systemic lupus erythematosus associated with thrombocytopenia: Case report. *Medicine (Baltimore)* (2021) 100(2):e24231.

10. Santacruz JC, Villota C, Ballesteros JG, Bello JM, Londoño JD. Subdural haematoma as the initial manifestation of systemic lupus erythematosus associated with severe pancytopenia. *Hematoma subdural como manifestación inicial de lupus eritematoso sistémico asociado a pancitopenia severaRevista Colombiana de ReumatologiaRev. Colomb. Reumatol.* (2023) 30(4):337. doi: 10.1016/j.rcreu.2021.08.005

11. Torné R, Rodríguez-Hernández A, Bernard T, Arikan Abelló F, Vilalta Castan J, Sahuquillo J. Subarachnoid hemorrhage in systemic lupus erythematosus: systematic review and report of three cases. *Clin Neurol Neurosurg* (2015) 128:17-24.

12. Owada T, Takahashi K, Kita Y. Subarachnoid hemorrhage in systemic lupus erythematosus in Japan: two case reports and a review of the literature. *Mod Rheumatol* (2009) 19(5):573-80.

13. Zhang Y, Liu SF, Zeng XJ. Subarachnoid hemorrhage due to systemic lupus erythematosus associated with multiple intracranial artery aneurysms. *Chin Med J (Engl)* (2019) 132(1):109-12.

14. Tsukamoto E, Tanei T, Senda J, Kato T, Naito T, Ishii K, et al. Subarachnoid Hemorrhage After Ischemic Stroke Associated with Systemic Lupus Erythematosus and Antiphospholipid Syndrome. *World Neurosurg* (2020) 136:248-52.

15. Song W, Li M, Zhu B, Chen W, Bao J, Wang D, et al. Spontaneous epidural hematoma complicated by systemic lupus erythematosus: one case report. *Int J Clin Exp Med* (2015) 8(9):16813-6.

16. Gauiran DTV, Lladoc-Natividad TEB, Rocha III, Manapat-Reyes BH. Seizure and Acute Vision Loss in a Filipino Lupus Patient: A Case of Posterior Reversible Encephalopathy Syndrome with Intraparenchymal Hemorrhage. *Case Reports in MedicineCase Rep. Med.* (2018) 2018. doi: 10.1155/2018/4238676

17. Aoki A, Kobayashi H. SYSTEMIC LUPUS ERYTHEMATOSUS WITH CEREBRAL HEMORRHAGE DUE TO AUTOIMMUNE ACQUIRED COAGULATION FXIII/13 FACTOR DEFICIENCY: A CASE STUDY AND LITERATURE REVIEW. *Ann Rheum Dis* (2023) 82:1499-500. doi: 10.1136/annrheumdis-2023-eular.651

18. Rangwala SD, Strickland BA, Rennert RC, Ravina K, Bakhsheshian J, Hurth K, et al. Ruptured Mycotic Aneurysm of the Distal Circulation in a Patient with Mucormycosis Without Direct Skull Base Extension: Case Report. *Oper Neurosurg (Hagerstown)* (2019) 16(3):E101-101E107.

19. Chattopadhyay P, Dhua D, Philips CA. Reversible diffuse neurological deficits in systemic lupus erythematosus: Report of a case. *LupusLupus* (2011) 20(10):1079. doi: 10.1177/0961203310396268

20. Goh KG, Ong SG. Recurrent spontaneous subdural hematoma secondary to immune thrombocytopenia in a patient with overlap syndrome. *Lupus* (2015) 24(1):90-3.

21. Yin R, Qiu C, Yin L, Zhang Y. Multiple spontaneous intracranial hemorrhages in a patient with systemic lupus erythematosus: a case report. *Int J Clin Exp Med* (2019) 12(8):10900-4.

22. Pahadiya HR, Lakhotia M, Gandhi R, Choudhary A, Madan S. Multiple intracranial hemorrhages in pregnancy: A common autoimmune etiology. *J Neurosci Rural Pract* (2016) 7(2):290-4.

23. Carreño-Tarragona G, Morales E, Jiménez-Herrero MC, Cortés-Fornieles E, Gutierrez E, Praga M. Lupus anticoagulant-hypoprothrombinemia syndrome: A rare association in systemic lupus erythematosus. *Síndrome de anticoagulante lúpico-hipoprotrombinemia: una extraña asociación en el lupus eritematoso sistémicoNefrologiaNefrologia* (2016) 36(2):186. doi: 10.1016/j.nefroe.2015.11.008

24. Llorente-Chávez A, 0000-0003-3399-506X AO, Plascencia-Félix JF, 0000-0003-1068-5253 AO, Merayo-Chalico J, 0000-0002-5870-0523 AO. Lupus anticoagulant-hypoprothrombinaemia syndrome: subdural haematoma as an unusual and initial manifestation. *Mod Rheumatol Case Rep* (2021) 5(2):306-9.

25. Boura P, Papadopoulos S, Tselios K, Skendros P, Dioritou O, Malamis G, et al. Intracerebral hemorrhage in a patient with SLE and catastrophic antiphospholipid syndrome (CAPS): Report of a case. *Clinical RheumatologyClin. Rheumatol.* (2005) 24(4):420. doi: 10.1007/s10067-004-1062-9

26. Perard L, Costedoat-Chalumeau N, Limal N, Hot A, Cohen J, Vauthier-Brouzes D, et al. Hemophagocytic syndrome in a pregnant patient with systemic lupus erythematosus, complicated with preeclampsia and cerebral hemorrhage. *Ann Hematol* (2007) 86(7):541-4. doi: 10.1007/s00277-007-0277-7

27. Lee JH, Lee SW, Choi CH, Ko JK. Does Systemic Lupus Erythematosus Increase the Risk of Procedure-Related Complication in Endovascular Treatment of Intracranial Aneurysm. *Yonsei Med J* (2020) 61(5):441-4. doi: 10.3349/ymj.2020.61.5.441

28. Khadka N, Kandel K, Mishra A, Jha S. Concurrent occurrence of acute pancreatitis and intracerebral hemorrhage as presenting manifestations in lupus: a case report. *Annals of medicine and surgery (2012)* (2023) 85(8):4067-70. doi: 10.1097/MS9.0000000000001009

29. Li J, Meng H, Jiang W, Liu J, Cui Z, Miao J. Cerebral venous sinus thrombosis and subdural hematoma in a female patient with systemic lupus erythematosus: a case report and literature review. *Ann Palliat Med* (2021) 10(7):8454-9.

30. Casanova Rivera MF, Ligua Duque NB, Moreno Veloz EA, Casanova Rivera PS. Cerebral Venous Thrombosis in a 17-Year-Old Female Patient: A Case Report. *Cureus* (2023) 15(7):e42384-42384e42384. doi: 10.7759/cureus.42384

31. Yin R, Qiu C-X, Yin L-J, Zhang Y. Case report multiple spontaneous intracranial hemorrhages in a patient with systemic lupus erythematosus: A case report. *International Journal of Clinical and Experimental MedicineInt. J. Clin. Exp. Med.* (2019) 12(8):10900.

32. Harriott A, Faye EC, Abreu N, Silverman S, Rordorf G. Aneurysmal Subarachnoid and Spinal Hemorrhage Associated With Systemic Lupus Erythematosus. *Stroke* (2016) 47(3):e42-5.

33. Bashir H, Ranganathan P. An Unusual Case of Cerebellar Hemorrhage in a Patient With Systemic Lupus Erythematosus. *Arthritis Care Res (Hoboken)* (2010) 62(5):738-42. doi: 10.1002/acr.20114

34. Ueno M, Nakano K, Yoshinari H, Nakayamada S, Iwata S, Kubo S, et al. An Autopsy Case with Cerebral Hemorrhaging due to disseminated Aspergillosis During Glucocorticoid Therapy for Overlap Syndrome of Systemic Lupus Erythematosus and Systemic Sclerosis. *Intern Med* (2019) 58(7):1023-7. doi: 10.2169/internalmedicine.1226-18

35. Sandouno TM, Bachir H, Alaoui HB, Hamaz S, Eloumri AA, Berrimi M, et al. Acute spontaneous subdural hematoma as an inaugural presentation of systemic lupus erythematosus with acquired factor XIII deficiency: a case report. *Pan Afr Med J* (2021) 39:207.

36. Stavropoulos I, Liverezas A, Papageorgiou E, Tsiara S. A rare case of heparin-induced thrombocytopenia and cerebral venous sinus thrombosis with antiphospholipid syndrome and possible systemic lupus erythematosus. *Aktualnosci NeurologiczneAktual. Neurol.* (2017) 17(2):121. doi: 10.15557/AN.2017.0013

37. Hui SC. A Rare Case of Bilateral Cerebral Hemorrhage due to Cerebral Vasculitis in an Elderly Lady with Systemic Lupus Erythematosus and Radiological Lung Malignancy. *Cerebrovasc Dis* (2023) 52:156-156.

38. Enescu CD, Basida B, Zalavadiya N, Akram R, Sarakbi H. A Diagnostic Dilemma: Catastrophic or Seronegative Antiphospholipid Syndrome. *Cureus* (2021) 13(10):e18745-18745e18745. doi: 10.7759/cureus.18745

39. Okumura E, Onuki H, Otsuka K, Sunaga S, Tanaka A, Jimbo H. A Case of Acquired Factor XIII Deficiency with Systemic Lupus Erythematosus Diagnosed after Repeated Intracerebral Hemorrhages. *NMC Case Rep J* (2023) 10:121-4.

40. Wu R, Hu S. A Case of "Refractory" Neuropsychiatric Lupus Responsive to Anticoagulation. *Case Rep Neurol Med* (2017) 2017:5726180-5726180. doi: 10.1155/2017/5726180
