# Supplementary material for: Case report and literature review: neuropsychiatric systemic lupus erythematosus presenting as massive intracerebral hemorrhage
Source: Front Immunol. 2026 Jun 9;17:1847320. doi: 10.3389/fimmu.2026.1847320 (PMC13286940; doi:10.3389/fimmu.2026.1847320)
Supplement: Supplementary file 3 [file Table3.docx]

**Table S3.** Summary of Clinical Studies on SLE with ICH (2005-2025)

| **Author, Year (Ref in manuscript)** | **Type of Study** | **Main Findings** |
| --- | --- | --- |
| Gao N, 2013 (4) | Retrospective cohort study | This single-center Chinese retrospective study of 6,553 admitted SLE patients found that intracranial hemorrhage, though rare (incidence 0.39%), carries a very high in-hospital fatality rate (23.1%) and is independently associated with thrombocytopenia (OR 3.687, independent risk factor), with headache being the most common presenting symptom (53.5%). |
| Arkema EV, 2017 (5) | Retrospective population-based cohort study | This Swedish population-based cohort study of incident SLE patients (n=3,390) found a twofold increased rate of ischemic stroke (HR 2.2), with the highest risk observed in females, those under 50 years, and notably within the first year following SLE diagnosis (HR 3.7), while the risk for intracerebral hemorrhage was not significantly elevated (HR 1.4). |
| Guraieb-Chahín P, 2020 (11) | Retrospective cohort study | This large single-center cohort study of 4,451 Hispanic SLE patients found a stroke prevalence of 3.1%. ICH accounted for 9.4% of all strokes (13/139). Its onset tended to occur later in the disease course, with 75% of cases occurring more than 3 years after SLE diagnosis, and 34% occurring more than 10 years after diagnosis. Hypertension was associated with 69% of ICH cases, representing a major contributing factor. |
| Huang JA, 2024 (12) | Retrospective population-based cohort study | This large population-based cohort study from Taiwan (8,310 SLE patients) found that the incidence of hemorrhagic stroke was significantly higher in SLE patients (1.5%) compared to non-SLE controls (0.6%), with more than a twofold increased risk (aHR = 2.24). Additionally, the use of antiplatelet agents in the SLE group was associated with an increased risk of hemorrhagic stroke (aHR = 1.74). |
| Yazdany J, 2020 (13) | Meta-analysis | In total, 26 studies were included. Patients with SLE have a significantly elevated risk of cerebrovascular events (CVEs), with a pooled RR of 2.18 for ischemic stroke, 1.84 for intracerebral hemorrhage, and 2.13 for composite stroke compared to the general population. |
| Holmqvist M, 2015 (14) | Meta-analysis | Total of 10 studies were included. Patients with SLE have a significantly elevated risk for all stroke subtypes, with a twofold higher risk for ischemic stroke (pooled RR 2.10), a nearly threefold higher risk for intracerebral hemorrhage (2.72), and an almost fourfold higher risk for subarachnoid hemorrhage (3.85) compared to the general population. |
| Bernatsky S, 2006 (15) | Retrospective population-based cohort study | This Canadian multi-site cohort study, with 10 centers and 2,688 patients, found a two-fold increased risk of mortality from cerebrovascular disease in SLE patients compared to the general population (SMR 2.0), with the highest risks observed for ill-defined cerebrovascular events (SMR 44.9) and other cerebrovascular disease (SMR 8.4), noting that deaths from cerebral hemorrhage appeared more common than those from infarction. |
| Hsu UH, 2024 (16) | Case-control study | This case-control study of young SLE patients (<50 years) in Taiwan identified hyperlipidemia (OR 19.36), antiphospholipid syndrome (OR 41.9), lower hemoglobin (OR 0.66), and higher SLEDAI-2k score (OR 1.22) as independent risk factors for cerebrovascular events, with hemorrhagic CVEs peaking within the first year of diagnosis and ischemic CVEs peaking during the 2nd to 5th year. |
| Rossides M, 2017 (17) | Nationwide registry cohort study | This Swedish nationwide registry study found that among individuals with first-ever stroke, those with SLE had significantly worse outcomes than those without SLE, including higher 1-year mortality after both ischemic (HR 1.85) and hemorrhagic (HR 2.30) stroke, and a nearly twofold increased risk of functional impairment at 3 months post-ischemic stroke (risk ratio 1.73). |
| Mok CC, 2009 (18) | Longitudinal cohort study | This longitudinal cohort study from Hong Kong (1999-2007) reported a mean annual cerebrovascular accident incidence of 6.45/1000 SLE patients, with a twofold higher overall risk compared to the general population (SIR 2.02). This risk was predominantly driven by ischemic stroke, which accounted for 90% of events, while hemorrhagic stroke comprised only 10% and occurred mainly in younger SLE patients. |
| Wang IK, 2012 (19) | Retrospective population-based cohort study | This large population-based cohort study from Taiwan, which included 13,689 patients with SLE, revealed that individuals with SLE had nearly three times the overall risk of stroke compared to controls (HR 2.90). The relative risk was most pronounced in the youngest age group (ages 1–17), with an HR of 163. Hemorrhagic strokes constituted a higher proportion of all strokes in the SLE cohort than in the non-SLE cohort (28.0% vs. 23.4%). Additionally, hospitalization costs associated with stroke were more than double for patients with SLE. |
| Chang KC, 2023 (20) | Retrospective population-based cohort study | This large Taiwanese population-based cohort study of 10,006 SLE patients demonstrated a clear association between disease severity and stroke risk, with those experiencing a severe flare having a dramatically higher risk of both ischemic (aHR 7.44) and hemorrhagic (aHR 22.49) stroke compared to SLE patients without severe flares. The graded relationship was evident in the crude incidences: hemorrhagic stroke occurred in 2.7% of the severe flare group, 1.3% of the non-severe flare group, and only 0.4% of controls. |
